# Survival Benefit of Intervention Treatment in Advanced Anaplastic Thyroid Cancer

**DOI:** 10.1155/2021/5545127

**Published:** 2021-06-03

**Authors:** Pornthep Kasemsiri, Pimpika Chaisakgreenon, Patravoot Vatanasapt, Supawan Laohasiriwong, Watchareeporn Teeramatwanich, Cattleya Thongrong, Teeraporn Ratanaanekchai, Surapol Suetrong

**Affiliations:** ^1^Department of Otorhinolaryngology, Srinagarind Hospital, Faculty of Medicine, Khon Kaen University, Khon Kaen, Thailand; ^2^Khon Kaen Head and Neck Oncology Research, Khon Kaen, Thailand; ^3^Department of Anesthesiology, Srinagarind Hospital, Faculty of Medicine, Khon Kaen University, Khon Kaen, Thailand

## Abstract

**Background:**

The management of anaplastic thyroid cancer (ATC) is controversial; thus, proper treatment and prognostic factors should be investigated.

**Objectives:**

To compare the survival outcomes of the intervention and palliative treatment in ATC patients.

**Methods:**

A hospital-based retrospective study was conducted at a single tertiary university hospital. The medical record charts were retrieved from November 20, 1987, to December 31, 2016. The final follow-up ended by December 31, 2017. The patients' demographic data, laboratory data, clinical presentation, and treatment modality results were analyzed.

**Results:**

One hundred twenty-one records were analyzed with a one-year overall survival rate of 3.5% (median survival time: 77 days); however, 16 cases had insufficient data to classify staging and treatment modalities. Therefore, 105 ATC patients (37 with stage IVa, 39 with stage IVb, and 29 with stage IVc disease) were included with a one-year overall survival rate of 4.0% (median survival time of 82 days). Intervention treatment allowed longer median survival times (*p* < 0.05) and a better survival rate (*p* < 0.05). Among the interventional treatment groups, postoperative chemoradiation yielded the longest median survival time (187 days) and the highest survival rate (20%) (*p* < 0.05). The intervention modality allowed a better median survival time at all stages, particularly in stage IVa (*p* < 0.05). Unfavorable prognostic factors were adjusted for in a multiple Cox regression model showing that significant factors included age ≥65 years (hazard ratio HR: 2.57), palliative treatment (HR: 1.85), and leukocytosis ≥10,000 cells/mm^3^ (HR: 2.76).

**Conclusions:**

Intervention treatment provided a better survival outcome in all stages, particularly in stage IVa, with a significantly better median survival time. Among interventional treatments, postoperative chemoradiation led to the longest survival rate, suggesting that this treatment should be considered in ATC patients with resectable tumors and no poor prognostic factors, such as older age and leukocytosis.

## 1. Introduction

Anaplastic thyroid cancer (ATC) is a rare disease. Although ATC only accounts for 1%–2% of all thyroid malignancies, it is a rapidly growing tumor with extremely aggressive behavior, accounting for more than 50% of all thyroid-related mortality [1–3]. Several studies have reported a median overall survival rate of less than 6 months and a 1-year survival rate of 20% [3–5]. Regarding ATC treatment, multimodality (including surgery, radiotherapy, and systemic therapy) is required for improved survival rates. The complete surgical removal of tumors in ATC is a good option for limited tumor invasion; however, most patients present with a rapidly enlarging mass [6–8]. Additionally, up to 70% of patients were reported to have aggressive ATC with invasion into surrounding tissues, including the muscle (65%), trachea (46%), esophagus (44%), and larynx (13%) [1]. Therefore, other interventional treatments were introduced to combine multimodality treatment to combat aggressive ATC. Sugitani et al. [9] reported that surgery and external beam radiotherapy ≥ 40 Gy were predictors of significantly better overall survival in any stage of ATC. Regarding systemic therapy, chemotherapy has been increasingly used over the last few decades [10]. Sugitani et al. [9] showed that chemotherapy was a predictor of significantly better overall survival for patients with stage IVB or IVC disease. Furthermore, novel systemic therapy (bovine serum ribonuclease [11], bone morphogenic protein [12], and p53 gene therapy [13, 14]) was proposed to alter the course of ATC. Therefore, the combination of multimodality treatment seemed to allow improvement in survival outcomes. However, these interventional treatments do not achieve universally beneficial outcomes; conversely, adverse side effects from interventional treatments may worsen the outcomes and make the poor survival rate even worse in patients with compromised health status. Therefore, interventional treatment should be reserved for patients with a good health status who can tolerate treatment side effects. For patients with a poor health status, supportive or palliative treatment should be considered to improve the quality of life and avoid the side effects of interventional treatments. However, survival rate data in patients with palliative treatment are lacking, as is comparative data assessing palliative care outcomes against the benefits of interventional treatments. Therefore, this study aimed to compare the survival outcomes from palliative versus intervention care and investigate unfavorable prognostic factors predictive of poor survival outcomes.

## 2. Materials and Methods

### 2.1. Study Design and Data Collection

A hospital-based retrospective study was conducted with anaplastic thyroid cancer patients at a single tertiary university hospital. The medical record charts from the ATC patients from November 20, 1987, to December 31, 2016, were retrieved. ATC was diagnosed based on fine-needle aspiration cytology and/or histopathology from the biopsy or surgical specimen. The patients' demographic data, laboratory data, clinical presentation, and treatment modality results were assessed. For the staging of ATC, we used the standard TNM classification of the 8th edition AJCC staging system. Regarding our treatment modality, total thyroidectomy was performed in patients with tumors localized at the thyroid gland, whereas thyroidectomy with extensive resection of the surrounding tissue was performed for patients with resectable extrathyroid invasion. Neck dissection at levels II to VI was performed in patients with clinical or cytopathological lymph nodes, while neck dissection at level VI was performed in patients with clinically negative cervical lymph nodes. Other modalities of treatment and radiotherapy were classified according to the total radiation dose. We allocated patients who received doses of more than 40 Gy to the intervention group and those with doses of less than 40 Gy to the palliative group. For the chemotherapy modality, we classified chemotherapy plus other therapy modalities (surgery and/or radiotherapy) as an intervention group, whereas a single chemotherapy modality was defined as the palliative group. The palliative group was reserved for patients with a tumor that was beyond surgery and poor health status and who were not candidates for definite radiotherapy. The follow-up time started from the date of the first treatment and ended by December 31, 2017.

### 2.2. Statistical Analysis

Statistical analysis was performed using STATA (v 10.0; Stata Corp., Texas, USA). Survival duration was analyzed using the days from the date of diagnosis to the date of death. Kaplan-Meier analysis was used to demonstrate the survival curve. Patients who were lost to follow-up or survived were considered censors. A comparison of the survival curves between the intervention and palliative group was performed using the log-rank test in each stage. Furthermore, univariate analysis was used for the Cox proportional hazard regression to identify significant prognostic factors. After that, the statistical significance of covariates on survival was adjusted with multiple Cox regression analysis to identify independent prognostic factors. *p* < 0.05 was considered statistically significant.

### 2.3. Ethics

The study was approved by the local ethics research committee (HE611221).

## 3. Results

### 3.1. Patient Demographics

The data for 121 patients with ATC (42 men and 79 women) were retrieved from the hospital database (Table 1). Almost half of the patients (40.5%) were in the 61- to 70-year age range. Most patients presented a tumor ≥5 cm (72.7%), and 11.6% showed extrathyroid invasion of vital structures, including the carotid sheath, subclavian artery, and intrathoracic structures.

### 3.2. Survival Data

**Figure 1:**
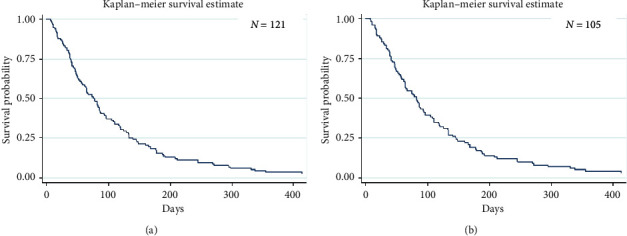
The 1-year overall survival rate and median survival time of all ATC patients were 3.5% (95% CI: 1.7–8.0) and 77 days (95% CI: 57–88), respectively (a). However, 16 ATC patients had insufficient data to classify the staging and modality of treatment. Thus, 105 ATC patients showed a 1-year overall survival rate of 4% (95% CI: 1.3–9.2) and a median survival time of 82 days (95% CI: 63–96) (b).

**Figure 2:**
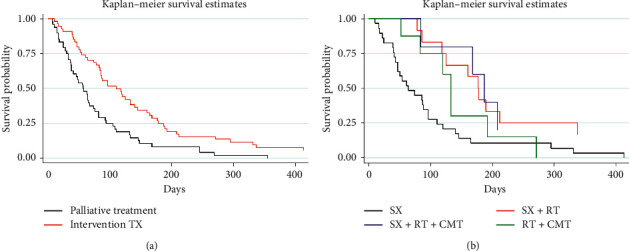
One hundred five ATC patients were classified, among whom 49 received palliative modality and 56 patients received interventional treatment. Intervention treatment allowed a median survival time of 110 days (95% CI: 84–140) that was better than palliative treatment. Palliative treatment allowed a median survival time of 58 days (95% CI: 38–74). Furthermore, the Kaplan-Meier survival curve was analyzed using the log-rank test, revealing that interventional treatment was significantly better than palliative treatment (*p*=0.0006) (a). In the interventional treatment, the combination of surgery with postoperative chemoradiation showed the best survival rate (log-rank test; *p*=0.01). The median survival time for interventional modalities was analyzed by subgroup and showed times of 187 days (95% CI: 84–208) in the surgery combined with postoperative chemoradiation treatment, 177 days (95% CI: 86–337) in the surgery combined with radiation treatment, 133 days (95% CI: 52–192) in the chemoradiation treatment, and 64 days (95% CI: 43–96) in the surgery-alone treatment (b).

**Figure 3:**
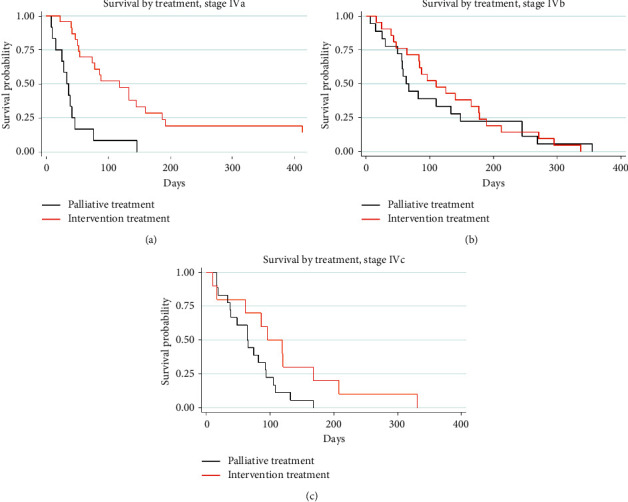
Thirty-seven ATC patients in stage IVa had a median survival time of 118 days (95% CI: 54–160) in the interventional treatment group, whereas the palliative treatment group had a median survival time of 33 days (95% CI: 10–46). This difference was statistically significant (*p* ≤ 0.001). The Kaplan-Meier curve showed the benefit survival rate in the intervention group (log-rank test; *p* ≤ 0.001) (a). In 39 patients with ATC stage IVb, the interventional treatment allowed a median survival time of 110 days (95% CI: 64–177), which was better than palliative treatment (median survival time: 63 days (95% CI: 49–133)); however, the median time survival difference was not statistically significant (*p*=0.63). The Kaplan-Meier curve showed that the intervention group seemed superior to palliative treatment but did not reach statistical significance (log-rank test; *p*=0.67) (b). Regarding the median survival time of 29 patients with ATC stage IVc, the intervention group (96 days (95% CI: 10–168)) was not significantly better than the palliative group (64 days (95% CI: 37–93)) (*p*=0.06). However, the Kaplan-Meier survival curve of the intervention group was not significantly better than that of the palliative group (log-rank test; *p*=0.055) (c).

The one-year overall survival rate of 3.5% (median survival time: 77 days (95% CI: 57–88)) with a median follow-up time of 74 days (range: 5–4,061 days) was observed in our 121 patients (Figure 1(a)); however, 16 ATC patients had insufficient data to classify TNM staging and treatment modalities. The remaining 105 ATC patients were classified as follows: stage IVa, 37 patients; IVb, 39 patients; IVc, 29 patients. The common pattern of regional cervical lymph node metastasis was a unilateral single node (19.8%), and the most common site of distant metastasis was the lung (22.6%). Regarding treatment modality, 49 ATC patients had received palliative treatment (35.2% supportive treatment and 11.4% palliative radiation), while 56 ATC patients received interventional treatment, including surgery alone (27.6%), chemoradiation (8.5%), surgery combined radiation (12.4%), and surgery combined chemoradiation (4.7%). The overall survival rate was 4.0% at 1 year (median survival time of 82 days (95% CI: 63–96)) in the 105 patients (Figure 1(b)). Comparing the interventional and palliative treatments, the overall median survival time of the interventional treatment (110 days) was almost twice as long and was significantly different (log-rank test; *p* < 0.05; Figure 2(a)) from that of the palliative treatment group (58 days). Among the interventional treatment groups, surgery with postoperative chemoradiation yielded the longest median survival time of 187 days and the longest survival rate of 20% (log-rank test; *p* < 0.05) (Figure 2(b)). The median survival time of intervention and palliative treatment was also compared in each stage. In stage IVa, the interventional treatment group (118 days (95% CI: 54–160)) had significantly longer survival than the palliative treatment group (33 days (95% CI: 10–46)) (*p* ≤ 0.001; Figure 3(a)); however, the median survival time of the interventional treatment group was not significantly longer than that of the palliative treatment group in stages IVb (intervention: 110 days (95% CI: 64–177); palliative: 63 days (95% CI: 49–133); *p*=0.63; Figure 3(b)) and IVc (intervention: 96 days (95% CI: 10–168); palliative: 64 days (95% CI: 37–93); *p*=0.06; Figure 3(c)).

### 3.3. Analysis of Factors Affecting Prognosis

Regarding prognostic factors, the univariate analysis found significantly poorer outcomes associated with an age ≥65 years (hazard ratio (HR): 1.6), palliative treatment (HR: 2.0), hypothyroidism (HR: 4.5), and leukocytosis (HR: 2.1) (Table 2). After that, these variables were adjusted for in the multivariate analysis, and an age ≥65 years (HR: 2.6), palliative treatment (HR: 1.9), and leukocytosis (HR: 2.8) were demonstrated to be significant independent variables for poorer outcomes (Table 3).

## 4. Discussion

Our results showed that most ATC patients were older than 60 years (73.6%) with a male to female ratio of 1 : 1.9, very similar to previous findings [15–17]. However, most of our ATC patients (72.7%) presented with tumors ≥ 5 cm in diameter, slightly larger than those reported in previous studies (53.0%–68.9%) [15, 18]. According to TNM staging, our patients were approximately equally distributed across each stage (35.2% for IVa, 37.1% for IVb, and 27.6% for IVc). In stage IVb and IVc cases with extrathyroid invasion, the tissue surrounding the thyroid gland was frequently involved, making complete removal challenging in patients with extensive involvement of vital structures. We found that 11.6% of our patients presented with tumors involving vital structures. Distant metastasis was also a prognostic factor for a poor survival outcome in 22.6% of patients in our series. Previous research reported ATC survival outcomes ranging from 2 to 10 months and > 2-year survival rates of 0%–10% [10], which were similar to our findings of the median survival time and 1-year survival rate of 77–82 days and 3.5%–4.0%, respectively. Survival outcomes in prior retrospective studies vary depending on the sample size, baseline demographic data, and selection bias. Our survival outcomes were likely worse than those of previous studies because of numerous cases with a large tumor involving vital structures and distant metastasis. Treatment modality has important effects on survival outcomes. Some studies advocate that multimodality treatment has benefits [19–21]; however, few studies report significant survival benefits from multimodality treatment [22, 23]. Our study found that interventional treatments provided better survival outcomes than palliative treatment (*p* < 0.05) in overall staging. However, the surgery and postoperative chemoradiation combination provided the best 1-year survival rate of 20.0% among the interventional treatment groups. These findings compare well with a previous study [21] that showed that complete ATC resection combined with postoperative adjuvant chemotherapy and irradiation resulted in longer-term survival, even with persistent minimal disease. Although interventional treatment seemed to provide superior survival outcome benefits, we also investigated possible differential effects across different staging levels. We found that intervention provided significantly better outcomes than palliative care in stage IVa (*p* < 0.05). Intervention treatment was also better than palliative care in stages IVb and IVc (*p* > 0.05) but not at a statistically significant level, possibly because of more aggressive tumors in these advanced stages.

Age, gender, tumor size, the extent of disease at presentation, acute symptoms, distant metastasis, leukocytosis, and multimodality therapy are previously reported prognostic variables associated with survival outcome [6, 7, 20, 21, 24–26]. In our study, the ATC patient prognosis mainly depended on age, leukocytosis, and treatment. Glaser et al. [27] reported that an age ≥ 65 years was an unfavorable prognostic factor. This finding was similar to our study finding that showed that older age was a factor for significantly higher mortality (HR: 1.55). Other authors have also reported that older age was a poor prognostic factor, but old age was variously defined. The range for old age was reported as ≥60–75 years in previous studies [25, 28, 29]. Furthermore, leukocytosis was observed to also predict poor survival outcomes. Jiang et al. [15] and Sugitani et al. [9] found HRs of 1.12 and 1.48, respectively. In our series, a white blood cell count ≥10,000/ml^3^ had a hazard ratio of 2.76 (*p* < 0.001) from the Cox regression analysis. This finding was comparable with that in previous reports investigating the effects of leukemoid paraneoplastic reaction by ATC tumor-secreted cytokines, including granulocyte-colony stimulating factor, granulocyte macrophage-CSF, and interleukin-6 [30, 31]. The final significant prognostic factor found in our study, treatment modality, revealed that palliative treatment predicted the poorest overall survival outcome, with an HR of 1.85 (*p* < 0.05). However, selection bias makes this finding unsurprising given that palliative care patients usually have advanced disease with high mortality.

Sugitani et al. [9] classified the modality benefits in each ATC staging and found that postoperative chemoradiation was a significantly favorable prognostic factor in stage IVb (HR: 0.45; *p*=0.083); however, in stage IVa, its benefits did not reach a statistically significant level (HR: 0.21; *p*=0.19). Although controversy persists concerning proper ATC treatment protocols, several previous studies suggest that multimodal treatment allows a longer survival rate. Kobayashi et al. [24] suggested active multimodality treatment at the early stage. The multimodality protocol of surgery and chemoradiation has been advocated as offering the highest survival rate [32–34]. In the present study, the combined modality of postoperative chemoradiation and radiotherapy led to longer median survival times of 187 days and 177 days, respectively, than surgery alone, which led to a survival rate of 64 days, again supporting the advantage of multimodality treatment in improving the survival outcomes. A negative prognostic association has been reported for hypothyroidism. Our study found that hypothyroidism was a negative predictor in the univariate analysis; however, the multivariate regression analysis showed that this difference was not statistically significant. Jiang et al. [15] found similar findings where the serum T4 levels were not statistically significant in Cox regression analysis. However, they observed that patients with low T4 levels had significantly lower survival rates than those with normal T4 levels. Several authors proposed that hypothyroidism may occur due to the tumor damaging the normal thyroid tissue [35, 36] and inhibition of changes in T4-to-T4 binding globulin by unsaturated fatty acids from hypoxic or injured tissue in severely ill patients [37]. Therefore, low T4 levels may represent a late stage of ATC with severe disease that indicates poor survival outcomes.

ATC is an extremely aggressive and rapidly progressing tumor that makes it difficult to use a randomized prospective protocol to evaluate treatment and survival outcomes; therefore, a retrospective chart review was selected as a feasible approach for this study. Although our study includes the limitations of retrospective studies, it showed that a multimodality treatment was superior to a palliative modality, particularly the combination of surgery and chemoradiation. Furthermore, we found that not only palliative treatment but also age and leukocytosis were unfavorable prognostic factors for predicting mortality outcomes. In the future, more laboratory information and detailed clinical data would allow for better investigation of prognostic factors.

## 5. Conclusion

The results obtained from the current study showed that interventional treatment led to better survival outcomes in all stages of ATC, particularly in stage IVa. Among interventional treatments, postoperative chemoradiation led to the longest survival rate and should be considered for ATC patients with a resectable tumor and no poor prognostic factors. Factors (including older age ≥ 65 years, leukocytosis ≥10,000 cells/ml^3^, and palliative treatment) should be considered as unfavorable predictive prognostic factors that may help to decide on the management of ATC.

## Figures and Tables

**Table 1:** Demographic data.

Characteristic	*N* (%)
Gender	
Female	79 (65.3)
Male	42 (34.7)

Age (years)	
≤40	3 (2.5)
41–50	6 (4.9)
51–60	23 (19.0)
61–70	49 (40.5)
≥70	40 (33.1)

Underlying disease	
No/Unknown	88 (72.7)
Diabetes mellitus	19 (15.7)
Hypertension	17 (14.1)
Dyslipidemia	3 (2.5)
Other	15 (12.4)

Thyroid function test	
Hypothyroid	8 (6.6)
Euthyroid	17 (14.1)
Hyperthyroid	3 (2.5)
Unknown	93 (76.9)

WBC (cells/ml^3^)	
≥10,000	48 (39.7)
<10,000	31 (25.6)
Unknown	42 (34.7)

Tumor size (cm)	
<5	6 (4.9)
≥5	88 (72.7)
Unknown	27 (22.3)
Extrathyroid invasion involved vital structures	14 (11.6)

Cervical lymph node metastasis	
No/Unknown	69 (57.0)
Unilateral single	24 (19.8)
Unilateral multiple	18 (14.9)
Bilateral	10 (8.3)

Distance metastasis	
No/Unknown	89 (71.8)
Lung	28 (22.6)
Bone	5 (4.0)
Liver	2 (1.6)

Staging	
IVa	37 (30.6)
IVb	39 (32.2)
IVc	29 (23.9)
Unknown	16 (13.2)

Treatment	
Supportive treatment	37 (30.6)
Palliative radiation	12 (9.9)
Surgery alone	29 (23.9)
Chemoradiation	9 (7.4)
Surgery combined radiation	13 (10.7)
Surgery combined chemoradiation	5 (4.1)
Unknown	16 (13.2)

**Table 2:** Unadjusted univariable Cox proportional hazard model of prognostic factors.

Variable	Hazard ratio (95% CI)	*p* value
Age (years)		
<65	Reference	0.022
≥65	1.6 (1.1–2.0)	

Treatment		
Intervention	Reference	0.001
Palliative	2.0 (1.3–3.0)	

Staging		
4a	Reference	
4b	0.95 (0.7–1.7)	0.819
4c	1.35 (0.92–2.27)	0.221

Thyroid function test		
Euthyroid	Reference	
Hypothyroid	4.50 (1.19–13.57)	0.008
Hyperthyroid	3.75 (0.93–15.07)	0.063

White blood cell (cells/ml^3^)		
<10000	Reference	
≥10000	2.05 (1.25–3.35)	0.004

Underlying disease		
Absent	Reference	
Present	1.19 (0.79–1.81)	0.403

Tumor size (cm)		
<5	Reference	
≥5	2.32 (0.84–6.38)	0.104

Extrathyroid extension		
No	Reference	
Yes	1.12 (0.75–1.69)	0.569

Cervical lymph node metastasis		
No	Reference	
Unilateral single	1.03 (0.64–1.65)	0.914
Unilateral multiple	0.76 (0.44–1.31)	0.328
Bilateral	1.41 (0.71–2.77)	0.323

Distance metastasis		
No	Reference	
Present (lung, bone, and liver)	1.33	0.183

**Table 3:** Adjusted multivariable Cox proportional hazard models of prognostic factors.

Variable	Adjust hazard ratio (95% CI)	*p* value
Age (years)		
<65	Reference	
≥65	2.6 (1.5–4.4)	0.001

Treatment		
Intervention	Reference	
Palliation	1.9 (1.1–3.1)	0.016

White blood cell (cells/ml^3^)		
<10000	Reference	
≥10000	2.8 (1.6–4.9)	<0.001

## Data Availability

The data that support the findings of this study are available from the corresponding author upon reasonable request.

## Abbreviations

ATC: Anaplastic thyroid cancer
AJCC: American Joint Committee on Cancer
HR: Hazard ratio.
